# 

**Published:** 1900-06

**Authors:** 


### The Library of the Bristol Medico-Chirurgical Society.

*The following donations have been received since the publication of the List in March:*

*May 31st, 1900.*

| | |
|---|---|
| L. M. Griffiths (1) | 4 volumes. |
| Manchester Medical Society (2) | 1 volume. |
| Trafford Mitchell, M.D. (3) | 6 volumes. |
| George Parker, M.D. (4) | 7 ,, |
| Arthur B. Prowse, M.D. (5) | 21 ,, |
| W. H. Spencer, M.D. (6) | 1 volume. |
| J. Taylor (7) | 1 ,, |

Unbound periodicals have been received from the Editor of *The Cincinnati Lancet-Clinic*, Messrs. Macmillan and Co., Dr. Arthur B. Prowse, and Dr. Shingleton Smith.

---

### THIRTY-SIXTH LIST OF BOOKS.

The titles of books mentioned in previous lists are not repeated.

The figures in brackets refer to the figures after the names of the donors, and show by whom the volumes were presented. The books to which no such figures are attached have either been bought from the Library Fund or received through the *Journal*.

**Barker, L. F.** ... *The Nervous System* ... 1899
**Bertram de Saint-Germain.** *Des Manifestations de la Vie et de l'Intelligence* (1) 1848
**Boorde, A.** ... *A Dyetary of Helth, 1568* ... 1870
**Brandt, G. H. and J. E.** *Royat-Medical Guide* ... (5) 4th Ed. [N.D.]
**Burghard, W. W. Cheyne and F. F.** *A Manual of Surgical Treatment.* Part III. ... 1900

**Buxton, D. W.** ... *Anæsthetics* .. ... ... ... ... ... ... ... 3rd Ed. 1900
*Catalogue of Books in the Library of the Bristol Naturalists' Society* ... (5) 1888
**Chapman, C. W.** *Heart Disease in Childhood and Youth* ... ... ... ... [1900]
**Cheadle, W. B.** *Lectures on the Practice of Medicine*... ... ... ... ... ... 1900
**Cheyne and F. F. Burghard, W. W.** *A Manual of Surgical Treatment.* Part III. ... . ... ... ... ... ... ... ... ... 1900
*Christison, Sir Robert, The Life of.* 2 vols. ... .. ... ... ... ... 1885-86
**Coats, J.** ... ... *Manual of Pathology.* 4th Ed. (Revised by L. R. Sutherland) ... ... ... ... ... ... ... ... ... 1900
**Cruveilhier, J.** *Traité d'Anatomie pathologique générale.* Tome I. (1) 1849
*Cyclopædia of Names, The Century* ... ... ... ... ... ... ... ... ... [1894]
**Davies, H.**... ... *The Young Wife's Guide* ... ... ... ... ... ... ... (4) 1852
**Dührssen, A.** ... *A Manual of Gynæcological Practice.* (Tr. by J. W. Taylor and F. Edge) ... ... ... ... 2nd Ed. 1900
**Emond, E.**... ... *Le Mont-Dore* ... ... ... ... ... ... ... 4e. Ed. 1900
*Encyclopædia Medica.* (Ed. by C. Watson) Vol. III. ... ... ... ... 1900
**Fenwick, S. and W. S.** *Ulcer of the Stomach and Duodenum* ... ... ... 1900
"*Festschrift*" *in Honor of Abraham Jacobi* ... ... ... ... ... ... ... 1900
**Gould, G. M.** ... *A Pocket Medical Dictionary* ... ... ... ... 4th Ed. 1900
**Grant, W.**... .. *On the Nature, Rise, and Progress of Fevers in London* (1) 1771
**Hall, Mrs. Charlotte** *Memoirs of Marshall Hall, M.D.* ... ... ... ... 1861
**Hartridge, G.** ... *Golden Rules of Ophthalmic Practice* ... ... ... ... [1900]
**Hawkins-Ambler, G. A.** *Gynæcological Nursing* ... ... ... ... ... ... 1900
**Hillier, A.**... ... *Tuberculosis* ... ... ... ... ... ... ... ... ... ... 1900
**Huxley, T. H.** ... *Lessons in Elementary Physiology* ... ... [5th Ed.] 1900
*Katalog over det Norske Mediciniske Selskabs Bibliotek—Tillægs No. II.* ... 1900
[**Keating, J. M.** (Ed.)] *Cyclopædia of the Diseases of Children.* Vol. V. 1899
**Kirkes, W. S.** *Hand-Book of Physiology.* 16th Ed., by W. D. Halliburton 1900
**Labat, A.** .. ... *Climat et Eaux minérales d'Angleterre* ... ... ... ... 1900
**Letchworth, W. P.** *Care and Treatment of Epileptics* ... ... ... ... ... 1900
**Lucas, J. J. S.** ... *Nordrach at Home* ... ... ... ... ... ... ... ... ... [N.D.]
**Maylard, A. E.**... *The Student's Handbook of the Surgery of the Alimentary Canal*... ... ... ... ... ... ... ... ... ... 1900
**Mayo, H.** ... ... *Outlines of Human Physiology*... ... ... (4) 4th Ed. 1837
**Mégret, A.** *Étude de Mensurations sur l'Homme préhistorique* ... ... (7) 1894
**Muir and J. Ritchie, R.** *Manual of Bacteriology* ... ... ... ... 2nd Ed. 1899
**Owen, Rev. R.** ... *The Life of Richard Owen.* 2 vols. ... ... 2nd Ed. 1895
**Paget, S.** ... ... *Experiments on Animals* ... ... ... ... ... ... ... 1900
*Pharmacopœia of the Leicester Infirmary* ... ... ... ... ... ... ... (5) 1897
**Phillips, R.** ... *Translation of the Pharmacopœia of the Royal College of Physicians, London,* 1836 ... ... (4) 2nd Ed. 1837
**Pollack, B.** ... *Methods of Staining the Nervous System.* (Tr. from 2nd German Edition by W. R. Jack) ... ... 1899
**Ritchie, R. Muir and J.** *Manual of Bacteriology*... .. ... ... 2nd Ed. 1899
**Robinson, T.** ... *The Diagnosis and Treatment of Syphilis* ... 2nd Ed. 1900
,, *The Diagnosis and Treatment of Eczema* ... 2nd Ed. 1900
**Robson, A. W. M** *Diseases of the Gall-Bladder and Bile-Ducts* ... 2nd Ed. 1900
**Ryan, M.** ... .. *A Manual of Midwifery* ... ... ... ... ...(4) 4th Ed. 1841
**Smith, E. N.** .. *Paralytic Deformities of the Lower Extremities* ... ... 1900
**Spencer, W. H.** *Consumption* ... ... ... ... ... ... ... ... ... (6) 1900
**Sternberg, M.** ... *Acromegaly* (Tr. by F. R. B. Atkinson) ... ... ... [1899]

Symes, J. O. ... *The Bacteriology of Every-Day Practice* ... ... ... ... 1900
Tirard, N. ... ... *Medical Treatment* ... ... ... ... ... ... ... ... ... 1900
Wallace, J. S. ... *Decay in Teeth* ... ... ... .. ... ... ... ... ... ... 1900

### TRANSACTIONS, REPORTS, JOURNALS, &c.

American Dermatological Association, Transactions of the, 1899 ... 1900
American Journal of Obstetrics, The ... ... ... ... ... ... ...Vol. XL. 1899
American Laryngological Association, Transactions of the, 1899 ... 1900
American Pediatric Society, Transactions of the ... ... ... Vol. XI. 1899
American Public Health Association, Reports and Papers of the Vol. XXV. 1900
Archives of Pediatrics, The ... ... ... ... .. ... ... Vol. XVI. 1899
Boston Medical and Surgical Journal, The ... ... ... ... Vol. CXLI. 1899
Bristol Medico-Chirurgical Journal, The ... ... ... ... Vol. XVII. 1899
Bristol Port Sanitary District--Annual Report of the Medical Officers of Health for 1899 ... ... ... ... ... ... ... ... ... ... ... ... 1900
British Journal of Dermatology, The ... ... ... ... ... ... Vol. XI. 1899
Brooklyn Medical Journal, The ... ... ... ... ... ... Vol. XIII. 1899
Centralblatt für Gynäkologie ... ... ... ... ... ... ... ... ... ... ... 1899
College of Physicians of Philadelphia, Transactions of the 3rd Ser., Vol. XXI. 1899
Écho médical du Nord, L' ... ... ... ... ... ... ... ... ... ... ... 1899
English Catalogue of Books, The, for 1899 ... ... ... ... ... ... ... 1900
Gazette des Hôpitaux ... ... ... ... ... ... ... ... ... ... ... ... 1899
Gazette des Hôpitaux de Toulouse ... ... ... ... .. ... ... ... 1899
Hospital—
St. Thomas's Hospital Reports ... ... ... ... ... Vol. XXVII. 1899
Journal d'Hygiène ... ... ... ... ... .. ... ... ... ... Vol. XXIV. 1899
Journal de Neurologie ... ... ... ... ... ... ... ... ... Tome IV. 1899
Journal of Cutaneous and Genito-Urinary Diseases ... Vol. XVII. [1899]
Journal of Mental Science, The ... ... ... ... ... ... Vol. XLV. 1899
Journal of Nervous and Mental Disease, The ... ... ... Vol. XXVI. 1899
Liverpool Medico-Chirurgical Journal, The ... ... ... Vol XIX. 1899
Llangyfelach Rural District Council—Annual Report on the Llandilo-Talybont Division. Six vols. ... ... ... ... ... ... (3) 1894–99
Local Government Board, Second Annual Report of the ... ... (1) 1873
Medical Annual, The ... ... ... ... .. ... ... ... ... ... ... 1900
Medical Chronicle, The ... ... ... ... ... 3rd Ser., Vol. II. 1899-1900
Münchener medicinische Wochenschrift ... ... ... ... ... ... ... 1899
Newquay, Eleventh Annual Report on the Health and Sanitary Condition of ... ... ... ... ... ... ... ... ... ... ... ... ... 1899
Nord médical, Le ... ... ... ... ... ... ... ... ... ... ...Tome V. 1899
Nursing Directory, Burdett's Official ... ... ... ... ... ... ... ... 1900
Philadelphia County Medical Society, Proceedings of the Vols. XVIII., XIX., 1897–98
Preparations], Annual Report [on New Medicinal ... ... (5) 1894–97; 1899
Progrès médical, Le ... ... ... ... ... ... 3e. Sér., Tomes IX., X. 1899
Progressive Medicine ... ... ... ... ... ... ... ... ... 4 vols. 1899
Recueil d'Ophtalmolgie ... ... ... ... ... ... ... ... ... ... (2) 1889
Revue de Thérapeutique médico-chirurgicale ... ... ... ... ... ... 1899
Royal Red Book, Webster's ... ... ... ... ... ... ... ... ... (4) 1878

St. Louis Medical and Surgical Journal, The Vols. LXXVI., LXXVII. 1899
Treatment ... ... ... ... ... ... ... ... ... ... ... ... ... Vol. III. 1900
Wiener klinische Wochenschrift ... ... ... ... ... ... ... ... ... ... ... 1899

---

### Obituary.

### Local Medical Notes.

### BRISTOL.

University College, Bristol.—Mr. F. R. Cross, M.B., F.R.C.S., has been appointed to the recently-instituted Lectureship of Ophthalmology.

At recent examinations Mr. W. S. V. Stock graduated as Bachelor of Medicine at the University of London, and Messrs. W. J. Lord and T. White were successful in obtaining the M.R.C.S. and L.R.C.P. diplomas.

Mr. J. Phillips, M.R.C.S., L.R.C.P., has been appointed Senior House Surgeon to the Bradford Royal Infirmary, and Mr. W. J. Lord, M.R.C.S., L.R.C.P., Assistant House Surgeon to the Bristol General Hospital.

Royal Infirmary.—The annual meeting of the subscribers and friends of this institution was held on March 27th, under the presidency of Sir Chas. D. Cave, Bart. The report stated that 3,087 in-patients and 39,883 out-patients had been treated during 1899, the numbers for 1898 being 2,969 and 40,504 respectively. The financial statement showed that the total ordinary income amounted to £11,479, against £9,913 in 1898, and the total ordinary expenditure to £14,393, against £14,218 in 1898. The committee state that the total deficit of the Infirmary is now £12,926, and add that unless more subscriptions are received they may be compelled to close some of the wards. Sir Charles Cave was re-elected president. The following scholarships and prizes had been awarded:—University Entrance Scholarship (£50), Mr. S. C. Hayman; Lady Haberfield Entrance Scholarship (about £30), Mr. L. W. Morris; Suple Medical Prize and Gold Medal, Mr. P. G. Stock; Tibbits Memorial Prize, Mr. D. T. Price; Special Midwifery Certificates, Mr. T. S. Davies, Mr. H. James, Mr. M. H. Phillips, Mr. C. F. Walters; Pathological Prizes, Mr. W. B. Prowse, Mr. P. G. Stock, Mr. W. N. Blatchford, Mr. H. Hemsted, Mr. E. M. Pearse, Mr. D. T. Price.

General Hospital.—The annual meeting of this institution was held on March 12th, under the presidency of Mr. W. Proctor Baker. The medical report stated that 2,023 in-patients had been admitted during 1899, against 1,393 in the previous year; the out-patients numbered 29,287, against 27,876 in 1898. The financial report showed that if it had not been for utilised legacies there would have been a deficit of £2,300; the workpeople's contributions amounted to £2,041. The ordinary finances showed an increase over 1898, but this was accounted for by the larger amount of donations. It was stated that the following scholarships had been awarded:—Clarke Surgical Scholarship of £15 to Mr. R. G. Johnson; Sanders Scholarship of £22 10s. to Mr. R. G. Johnson; Lady Haberfield prize of £29 9s. 8d. to Mr. L. W. Morris; Martyn Memorial Scholarships, £10 to Mr. T. Aubrey and £5 each to Mr. R. G. Johnson and Mr. L. A. Moore.

Bristol City and County Lunatic Asylum.—The thirty-ninth annual report of this institution, which has just been issued, shows that on December 31st, 1899, there were 778 patients—379 males and 399 females—in the asylum. The highest number resident at any one time in the year was 799. The extension of the asylum buildings, with the exception of the isolation hospital, will shortly be completed, and will be ready for occupation early in the summer. The committee, in concluding the report, expressed their high appreciation of the manner in which the medical superintendent and the officers and staff of the asylum had performed their respective duties during 1899.

Royal Hospital for Sick Children and Women.—At the annual meeting, held on March 17th, the medical report stated that the out-patients numbered 4,978, and that 690 children and 45 women were admitted during the year; 443 dental cases had been attended to. The financial statement showed that the ordinary expenditure of the year amounted to £4,422, and the ordinary income was £3,657, leaving a deficiency of £765. The past year commenced with an adverse balance of £2,811, towards which £889 had been received by special donations and £1,200 had been transferred from the legacies account, so that at present there is a total deficit of £1,487.

Eye Hospital.—The eighty-ninth annual meeting of the Governors of this institution was held on March 28th, under the presidency of Mr. F. Richardson Cross. The medical report states that 364 in-patients and 4,975 out-patients had been treated during the year. The financial statement showed that the ordinary income amounted to £1,022, and there was now an adverse balance of £499. The Committee reported that a new story was being added which would provide better sleeping accommodation for nurses and servants, some small wards for patients, and a good operating theatre. A new wing was also being built, which would contain a sitting-room for nurses and a new operating theatre for out-patient cases. The approximate cost of the alterations will be about £6,000, towards which £2,500 have been received from private collections, and the committee appeal to the public for further subscriptions.

Eye Dispensary.—The annual meeting of the subscribers and friends of this institution was held on April 3rd, under the presidency of Mr. P. J. Worsley. The medical report showed that 1,762 patients had been treated during 1899, against 1,710 in 1898. The financial statement showed a favourable balance of £8.

Port Sanitary District.—The annual report of the medical officers of health of the Port Sanitary District for 1899 has just been issued. It shows that 1,544 vessels were inspected in dock during the year, and that the percentage of these found with sanitary defects was 14.4 as against 17.1 in 1897, the previous lowest record. Three notifications of suspected cases of plague were received from London, Southampton, and Cardiff respectively. Disinfection was carried out on five vessels from infected ports, especial efforts being made to exterminate the rats on board.

Medical Missionary Society.—The twenty-eighth annual meeting of this Society was held on March 13th, under the presidency of Mr. J. Storrs Fry. The honorary secretary stated that 7,535 patients had been under treatment during 1899; their attendances numbered 22,587. 1,833 patients had been attended at their homes. The financial report showed that the adverse balance of 1898 had been slightly reduced by a legacy received, but the expenditure exceeded the income from subscriptions and donations.

Hospital Sunday.—At the meeting of the Committee of the Bristol Hospital Sunday Fund held recently, it was stated that the total collections received amounted to £1,362. There were 200 collections in churches and chapels, being 49 in excess of last year.

Medical Dramatic Club.—The members of this Club celebrated their twenty-third anniversary by the production in April of Mr. J. M. Barrie's comedy "The Professor's Love Story." A collection was made on behalf of the Royal Infirmary and General Hospital.

Princess Christian Hospital.—News was received in Bristol recently that Major Mathias, R.A.M.C., and Mr. Paul Bush have accepted provisionally, on behalf of Mr. Mosely (who fitted out the Princess Christian Hospital), the generous offer by the owner of the large estate of Pinetown as a site for the hospital. The place is situated between Pietermaritzburg and Durban. The whole estate has been placed unreservedly at the disposal of the hospital staff. It is known in the district as "the garden of Natal." It lies 1,000 feet above sea level, and has an excellent water supply. The hospital will be in close relationship with the Princess Christian Hospital train, which will convey the injured and sick to the doors of the hospital buildings.

### BATH.

Royal Mineral Water Hospital.—The annual meeting of this institution was held on May 1st, under the presidency of Colonel Vaughan-Dymock. The report stated that 1,251 in-patients had been admitted during 1899, the daily average number in the hospital was 152, and the average stay of each patient was 44¾ days. The financial statement showed that the expenditure amounted to £5,879, which was higher than usual, owing to considerable repairs and improvements having been made in the hospital; there was a favourable balance of £464 remaining. Twenty beds have been placed at the disposal of the War Office authorities for the wounded soldiers, but up to the present only nine soldiers have been sent.

### BURNHAM.

Public Health.—Dr. F. C. Berry has been re-appointed Medical Officer of Health.

### CARDIFF.

Infirmary.—The annual meeting of those interested in this institution was held on February 28th, under the presidency of General Lee. The annual report stated that during 1889 there had been 1,700 in-patients admitted. The out-patients numbered 13,556, an increase of 513 as compared with the preceding year. The financial statement showed that the ordinary income amounted to £6,988, an increase of £98 as compared with 1898. Legacies and donations amounted to £2,600, and the subscriptions of working men had increased by £300. The total expenditure was £9,050, an increase of £520 as compared with the previous year. The chairman stated that the adverse balance of the Infirmary was now about £8,000.

Dr. W. G. Williams has been appointed Assistant House Surgeon.

### CHELTENHAM.

General Hospital.—The annual meeting of the subscribers and friends of this institution was held on March 21st, under the presidency of Colonel Croker-King. The medical report stated that 804 in-patients and 4,771 out-patients had been treated during the year, and at the branch dispensary 3,835 patients had been attended. The financial statement was satisfactory, but showed that although subscriptions had increased, donations and legacies had fallen off. A new wing had recently been added to the hospital, and a room fitted up for Röntgen ray work, and an ophthalmic room provided. The average cost of each in-patient was £4 14s. 2d., and of each out-patient 3s. 5½d. Colonel Croker-King was re-elected president.

### DEVONPORT.

The Military Hospital.—The work of erecting the new buildings of the Military Hospital, Stoke, is already well advanced; one of them is ready for occupation, and the remainder will soon be finished. The new buildings, or huts, of which there will be nine, are being erected in portable sections, so that they can be removed to another site if required. Notwithstanding this feature of their construction, they are of a substantial character, and internally resemble in every respect an ordinary hospital ward. Each hut will be 70 feet long with a breadth of 20 feet 7 inches, and will accommodate sixteen patients, with separate rooms for attendants and other purposes. The system of ventilation adopted is that by which a revolving ventilating tube will run the whole length of the roof.

### EXETER.

Royal Devon and Exeter Hospital.—The annual meeting of this institution was held on February 22nd, under the presidency of Colonel Lucas. The medical report stated that 1,322 in-patients had been admitted, against 1,343 in 1898. The out-patients numbered 4,303, against 3,850 in the preceding year. Seventy-five patients had been sent to the Ockenden Convalescent Home in connection with the hospital. The average daily number of in-patients was 145. The financial statement showed that the income from all sources amounted to £13,698; the ordinary expenditure was £8,460, and the extraordinary expenditure was £3,677, making a total of £12,137.

### PLYMOUTH.

Public Health. — The Borough Council has decided to add measles and whooping-cough to the list of diseases to be notified under the Infectious Diseases Act. It has further determined that phthisis should be made notifiable at the discretion of the medical practitioners. It has also decided to enlarge the lunatic asylum at Blackadown by erecting buildings to accommodate 250 additional patients. The estimated cost was £32,500. A tender has been accepted for erecting sewage works at a cost of £88,026.

Prevention of Tuberculosis.—On April 19th the Devon and Cornwall Branch of the National Association for the Prevention of Consumption held its annual meeting at Plymouth. The report of the committee gave some interesting information as to the existence of tuberculosis in dairy cattle in the West. Out of 204 cows in the Ilfracombe district, only one was found to be tuberculous. As regards the question of notification, the sanitary committees of Devonport and Stonehouse are considering the question of voluntary notification.

### NEWQUAY.

Public Health.—At a meeting of the Urban District Council held on May 1st, the Medical Officer of Health (Dr. A. Hardwick) reported that 149 cases of measles had been notified during April. After a considerable discussion the Council decided to ask the Local Government Board to consent to measles being excluded from the list of notifiable diseases in Newquay.

### TORQUAY.

Torbay Hospital.—The annual meeting of this institution was held on February 15th, under the presidency of Mr. R. Mallock. The medical report showed that 359 in-patients had been admitted, against 366 in 1898. The expenditure amounted to £2,127, and there was an adverse balance of £382. The committee decided that the honorary medical staff should form a council for the consideration of all professional subjects connected with the hospital, and that all by-laws, etc., proposed by them should be subject to revision and confirmation by the board of management.

### WESTON-SUPER-MARE.

Royal West of England Sanatorium.—The annual meeting was held on February 22nd, under the presidency of Mr. F. J. Fry, J.P. The medical report showed that 2,117 patients had been admitted (1,171 men and 946 women), showing an increase of 114 on the number for 1898. 8,450 hot and cold sea-water baths had been used. The income for the year amounted to £3,585, and the expenditure £3,430. The committee have placed at the disposal of the Government, free of all charges, one of the principal wards for the use of convalescent soldiers returned from South Africa, and already four of them are in the sanatorium.

